# BanLec-eGFP Chimera as a Tool for Evaluation of Lectin Binding to High-Mannose Glycans on Microorganisms

**DOI:** 10.3390/biom11020180

**Published:** 2021-01-28

**Authors:** Zorana Lopandić, Luka Dragačević, Dragan Popović, Uros Andjelković, Rajna Minić, Marija Gavrović-Jankulović

**Affiliations:** 1 Department of Biochemistry, Faculty of Chemistry, University of Belgrade, 11000 Belgrade, Serbia; lopandic93@gmail.com; 2 Institute of Virology, Vaccines and Sera, 11152 Belgrade, Serbia; lukadragacevic@gmail.com (L.D.); minicrajna@gmail.com (R.M.); 3 Institute of Chemistry, Technology and Metallurgy, National Institute of the Republic of Serbia, University of Belgrade, 11000 Belgrade, Serbia; dpopovic@chem.bg.ac.rs (D.P.); uros@chem.bg.ac.rs (U.A.); 4 Department of Biotechnology, University of Rijeka, 5100 Rijeka, Croatia

**Keywords:** banana lectin, eGFP, fluorescence, viral glycoproteins, influenza vaccine, florescence-linked lectin sorbent assay, *Salmonella* strains

## Abstract

Fluorescently labeled lectins are useful tools for in vivo and in vitro studies of the structure and function of tissues and various pathogens such as viruses, bacteria, and fungi. For the evaluation of high-mannose glycans present on various glycoproteins, a three-dimensional (3D) model of the chimera was designed from the crystal structures of recombinant banana lectin (BanLec, Protein Data Bank entry (PDB): 5EXG) and an enhanced green fluorescent protein (eGFP, PDB 4EUL) by applying molecular modeling and molecular mechanics and expressed in *Escherichia coli*. BanLec-eGFP, produced as a soluble cytosolic protein of about 42 kDa, revealed β-sheets (41%) as the predominant secondary structures, with the emission peak maximum detected at 509 nm (excitation wavelength 488 nm). More than 65% of the primary structure was confirmed by mass spectrometry. Competitive BanLec-eGFP binding to high mannose glycans of the influenza vaccine (Vaxigrip^®^) was shown in a fluorescence-linked lectin sorbent assay (FLLSA) with monosaccharides (mannose and glucose) and wild type BanLec and H84T BanLec mutant. BanLec-eGFP exhibited binding to mannose residues on different strains of *Salmonella* in flow cytometry, with especially pronounced binding to a *Salmonella* Typhi clinical isolate. BanLec-eGFP can be a useful tool for screening high-mannose glycosylation sites on different microorganisms.

## 1. Introduction

Lectins are carbohydrate-binding proteins involved in many biological processes like recognition and binding of glycans, host-pathogen interactions, cell targeting, cell-cell communication, induction of apoptosis, cancer metastasis, and differentiation [1,2]. Many different lectins are known and their glycan-binding specificities characterized (consortium for functional glycomics at http://www.functionalglycomics.org/). Antimicrobial activities of lectins toward viruses, fungi, and bacteria have been reported [3], with plant lectins as the well-characterized group.

The first report on the isolation of banana lectin (BanLec) was from *Musa paradisiac* [4]. It belongs to the jacalin-related superfamily (mJRL) of lectins, which recognizes and binds to high mannose glycans [5]. It is homodimeric in solution, with a molecular mass of about 26 kDa. Each subunit consists of twelve β-strands arranged in a β-Prism-I fold [6,7]. BanLec is an IgG4-inducing antigen [8] and a potent inhibitor of human immunodeficiency virus (HIV) replication [9,10]. BanLec binds to the glycosylated proteins in the viral envelope and inhibits its cellular entry by blocking glycan-mediated interactions with the CD4 receptor and CCR5/CCR4 co-receptors on host cells [10]. Because of its ability to induce T cell proliferation, BanLec has also been recognized as a potential immunomodulatory molecule [11,12,13]. It was shown that mutation of the amino acid at position 84 from histidine to threonine minimizes the wild-type banana lectin’s mitogenicity, without compromising antiviral activity against viruses that have high-mannose-type N-glycans on their surfaces [14].

Fluorescently labeled lectins are useful tools for both in vivo and in vitro study of the structure and function of tissues [15,16,17], pathological processes, including cancer [18,19,20], as well as those initiated with pathogens such as viruses, bacteria, and fungi [21].

Lectins can be fluorescently labeled using a chemical approach. However, chemical labeling can change the secondary and tertiary structure of lectins and consequently impair their interaction with glycans [22]. The binding constant between lectins and glycans is usually low, and every change of lectin structure can introduce a change of binding site and potentially affect the binding constant.

Green fluorescent protein (GFP) is a ready-made fluorescent polypeptide that finds broad application in various assays and in vivo imaging. The most explored application is a genetic fusion with a protein of interest [23]. The fused protein preserves its function along with the acquired fluorescent property of the GFP expressing gene. For instance, a neurocan–GFP which specifically binds to hyaluronan was employed for revealing a detailed picture of hyaluronan tissue distribution [24], while eGFP-Nictaba, the cytoplasmic/nuclear tobacco lectin, can interact with high-mannose and complex N-glycans [25]. Fluorescently labeled *Aspergillus oryzae* lectin is useful tool for the detection of the α(1,6)-fucose attached to the core N-glycan (core fucose) of glycoproteins known to play essential roles in various pathophysiological events [26].

In this study, we have designed in silico, produced, purified, and characterized recombinant BanLec-eGFP chimera. The application of BanLec-eGFP was tested with competitive florescence-linked lectin assay (FLLA) for studies of interactions between the lectin and high-mannose carbohydrates present on the trivalent inactivated A and B split-virion influenza vaccine; and with flow cytometry for the detection of high mannose content lipopolysaccharides (LPS) on the surface of bacteria.

## 2. Materials and Methods

### 2.1. Strains and Plasmids

*Escherichia coli* strain DH5α (Invitrogen Thermo Fisher Scientific, Carlsbad, USA) was used as a host to propagate plasmids pUC57-eGFP (GenScript HK Limited, Hong Kong, China) and BanLec gene-containing pET-23b, pET-23b-BL. Bacteria were grown in Luria-Bertani (LB) medium (10 g/L Tryptone (Torlak, Institute of Virology, Vaccines, and Sera, Belgrade, Serbia), 5 g/L NaCl (Betahem, Belgrade, Serbia), 5 g/L yeast extract (Torlak), and 15 g/L agar (Torlak)) supplemented with antibiotics when appropriate.

### 2.2. Design, Cloning of eGFP in pET-23b-BL

The three-dimensional (3D) model of the BanLec-eGFP chimeric structure is designed from the crystal structure of a recombinant banana lectin, Protein Data Bank (PDB) entry 5EXG [10] (chain A containing 141 amino acids) and the crystal structure of an enhanced green fluorescent protein, PDB entry 4EUL [27] (chain A containing 239 amino acids) by applying the molecular modeling and molecular mechanics to optimize the structure. All modifications in the sequence of BanLec-eGFP chimera relative to the used crystal structures, such as replacing the residues, removing the excessive, and adding the missing residues, were done in Discovery Studio 19.1.0. [28] The N-terminal part (MVS) and C-terminal fragment (GMDELYK) were modeled in a random coil conformation but ended up with a few turns and bends.

A glycine-serine-glutamate-phenylalanine (GSEF) linker was constructed between the BanLec and eGFP to prevent steric obstacles. The GSEF linker was modeled as a coil extension to the *β*-strand secondary structure of the C-terminus of eGFP in Discovery Studio 19.1.0 [28]. The computer-generated BanLec-eGFP chimeric design was obtained by connecting the three fragments and adding the hydrogen atoms, followed by the full structural minimization in NAMD 2.9 [29] with CHARMM22 force field [30] in implicit water model for 300 ps. The optimized resulting structure was strain-free, fully minimized, and relaxed without any bond, angle, or torsion angle restraints, and van der Waals atomic clashes. The final BanLec-eGFP structure comprised 384 amino acids, which encode for the BanLec, linker GSEF, and enhanced green fluorescent protein (eGFP). The strategy of cloning eGFP in the vector that already contains the BanLec gene sequence was conducted using *Nde*I (Thermo Fisher Scientific, Waltham, MA, USA) and *Bam*HI (Thermo Fisher Scientific) restriction enzymes.

### 2.3. Expression of BanLec-eGFP

*Escherichia coli* BL21 (DE3)-pLysS (Agilent Technologies Inc., La Jolla, CA, USA) strain was employed as host for the expression of recombinant chimera. Bacteria were grown in LB medium containing 100 μg/mL of ampicillin and 25 μg/mL of chloramphenicol until optical density (OD) at 600 nm of 0.6–0.8 was reached. The BanLec-eGFP synthesis was induced by adding isopropyl-D-thiogalactopyranoside (IPTG) (VWR International, LLC, Radnor, PA, USA) at a concentration of 1 mM, following incubation for 5 and 12 h at 25 °C. After expression, the cell pellet obtained by centrifugation (3000× *g* for 20 min) was resuspended in 150 mM NaCl, 20 mM tris(hydroxymethyl)aminomethane (TRIS) HCl pH 7.6, and sonicated by the ultrasonic horn. The supernatant obtained by centrifugation (3000× *g* for 20 min) was used for downstream processing of the expressed chimera.

### 2.4. Purification of Recombinant BanLec-eGFP

BanLec-eGFP was purified by combining affinity chromatography on branched α-1,6 and α-1,3 glucan polymer (Sephadex G-75 superfine, column volume (CV) = 10 mL) and ion-exchange chromatography (HiTrap ANX column, 1 mL, GE Healthcare, Little Chalfont, UK). After pre-equilibration with 20 mM TRIS HCl pH 7.6, 150 mM NaCl (TRIS buffered saline, TBS), the matrix (10 mL) was incubated with the protein solution at room temperature for 1.5 h. After the transfer to a column and thorough washing with five column volumes (CV) of TBS, bound BanLec-eGFP was eluted from the matrix with 0.3 M glucose in TBS and collected in tubes (1.5 mL).

After affinity chromatography, collected samples were purified by ion-exchange chromatography connected to Äkta Purifier (GE Healthcare) using a combination of equilibration buffer (10 mM TRIS HCl, pH 8.0) and elution buffer (10 mM TRIS HCl, 0.5 M NaCl, pH 8.0), at room temperature and a flow rate of 1 mL/min. The column pressure limit was 0.3 MPa. Bound proteins were eluted using a linear gradient of elution buffer (0–100% (*v*/*v*), for 10 column volumes). The BanLec-eGFP was eluted at 50% of the elution buffer.

The wild type BanLec (GenBank accession number EU0556441) and BanLec H84T mutant were produced and purified according to the same protocol as BanLec-eGFP.

After purification, BanLec-eGFP protein was analyzed by Western blot. After blocking the membrane with 5% skim milk in TBS with 0.05% Tween 20 (tTBS) for 1 h, polyclonal rabbit anti-rBanLec antibodies [31] (dilution 1:10,000) were added, and the period of incubation was 1 h. The membrane was washed, and alkaline phosphatase labeled goat anti-rabbit IgG (Sigma-Aldrich, St. Louise, MO, USA) was added. The binding of labeled antibodies for antigen was detected with nitro blue tetrazolium chloride/5-Bromo-4-chloro-3-indolyl phosphate (Serva, Heidelberg, Germany).

### 2.5. Analysis of the BanLec-eGFP Structure by CD Spectroscopy, Fluorescent and Mass Spectrometry

For assessing the secondary structures of the recombinant BanLec-eGFP, Circular dichroism (CD) spectra were recorded at 25 °C on Jasco J-815 circular dichroism spectropolarimeter (JASCO Inc., Easton, PA, USA) in the wavelength range from 185 to 260 nm in a cuvette with a path length of 0.1 mm. The CD data were collected in intervals of 0.1 nm, at a velocity of 50 nm/min. BanLec-eGFP was dialyzed against 20 mM TRIS HCl, pH 7.6 before spectral analysis. Purified BanLec-eGFP (0.92 mg/mL, 80 µL) was used for the study.

The fluorescence of BanLec-eGFP was measured using a spectrofluorimeter FLUOROMAX-4 Jobin Yvon (HORIBA, Kyoto, Japan). The emission spectra were recorded between 400–650 nm. The concentration of BanLec-eGFP used for the analysis was 0.92 mg/mL in 20 mM TRIS HCl buffer, pH 7.6.

To confirm the amino acid sequence, BanLec-eGFP was analyzed by mass spectrometry. The protein sample was separated by SDS-PAGE, followed by Coomassie Blue staining of the gel. The protein band corresponding to BanLec-eGFP was excised and destained by 100 mM NH_4_HCO_3_/50% acetonitrile (ACN). The gel pieces were then reduced for 40 min by 20 mM dithiothreitol and subsequently alkylated with 50 mM iodoacetamide for 30 min in the dark. The samples were digested with sequencing grade trypsin according to the manufacturer’s instructions (Promega, Mannheim, Germany). Before MS analysis, samples were cleaned over the C18 zip tip (Millipore, MA, USA). For MS analyses of the tryptic fragments, α-cyano-4-hydroxycinnamic acid as a matrix was used. Tryptic maps were obtained on matrix assisted laser desorption ionization-time of flight/time of flight (MALDI-TOF/TOF) Ultraflextreme mass spectrometer (Bruker) operating in a reflectron positive mode and a peptide calibration standard mixture (Bruker 206195) was used for mass calibration.

The obtained peptide masses were searched against the NCBI protein sequence database using the MASCOT program (http://www.matrixscience.com/search_form_select.html).

### 2.6. Competitive FLLSA (Fluorescence-Linked Lectin Sorbent Assay)

Recombinant BanLec-eGFP chimera was employed in competitive FLLSA for the influenza virus detection to test its applicability in high-mannose binding assays. The assay was performed by inhibiting the BanLec-eGFP binding to the immobilized hemagglutinin from a Vaxigrip™ vaccine (Sanofi Pasteur, Lyon, France) with monosaccharides (mannose or glucose), wild type BanLec, and BanLec H84T mutant. Black 96-Well Immuno plates (Thermo Fisher Scientific) were coated with hemagglutinin (0.5 μg per well) in 15 mM Na_2_CO_3_/35 mM NaHCO_3_, pH 9.5 overnight at 4 °C. Plates were washed with tPBS (phosphate buffered saline, PBS with 0.05% Tween 20). Then, the plates were blocked with 3% bovine serum albumin (BSA) in tPBS for 1 h at 37 °C. After washing, BanLec-eGFP (50 μg per well) was mixed without (control) or with different amounts of monosaccharides (0.01, 0.1, 1, 10, 100, 1000, 5000 mM) or BanLec inhibitors (BanLec wt, or BanLec H84T; 0.01, 0.1, 1, and 10 μg per well), which were added to the wells, and incubated in the dark for 2 h at room temperature. The FLLSA was performed in triplicate and the emission was measured at 535 nm on a fluorescent ELISA reader. Percent of inhibition was calculated as

(1) $$\%\;\mathrm{of}\;\mathrm{inhibition}\;=\;100\;\times \;(1\;-\;\frac{\left(X-\min\right)\;}{\left(\max-\min\right)})$$

and IC50 value was determined as a concentration of inhibitor at 50% of inhibition.

### 2.7. Assessment of Fluorescence-Linked Lectin Binding to Bacterial Cells with Flow Cytometry

For the assessment of recombinant BanLec-eGFP binding to bacterial cells, 7 strains of proteobacteria, species *Salmonella enterica*, subspecies *enterica* were used. The bacteria used were: serovar Enteritidis ATCC 13076; serovar Enteritidis clinical isolate E; serovar Typhimurium isolate B; serovar Typhimurium ATCC 14028; serovar Typhimurium isolate 2865; serovar Typhi clinical isolate 1243; and serovar Typhi clinical isolate 12. The bacteria were cultivated in nutritious broth for 24 h and washed twice in PBS, by centrifugation at 2000× *g* for 15 min. Cell density was adjusted to be the same for all strains, giving optical density of 0.15 at 620 nm in a 100 µL volume in ELISA plate. The staining with BanLec-eGFP was done by aliquoting the bacteria and adding BanLec-eGFP. Incubation period was 15 min, at 4 °C, in the dark; followed by washing with PBS and centrifugation 2000× *g* for 15 min two times. Finally, the bacteria were resuspended in PBS with 0.4% formaldehyde (PBS-FA).

Inhibition with monosaccharides was performed on serovar Typhimurium isolate 2865. The bacterial suspensions were centrifuged for 15 min 2000× *g* and PBS was removed. The bacteria were vortexed and different amounts of monosaccharides were added. This was followed by the addition of 1 µg BanLec-eGFP. After incubation for 15 min, at 4 °C, in the dark, the bacteria were washed twice by adding PBS, centrifuged as before, and resuspended in PBS-FA. Each concentration point was done in quadruplicate.

The staining with BanLec-B (biotin labeled BanLec) was done the same way, except that after washing the unbound BanLec-B 0.2 µg streptavidin-phycoerythrin (PE) (eBioscience) was added and after 15 min of incubation, the bacteria were washed as before.

The signal was analyzed with FACSVerse with BD FACSuite software (BD Biosciences). The graphs were drawn and results analyzed with OriginPro 8 and GraphPad Prism software.

## 3. Results

### 3.1. Design, Cloning, Expression, and Purification of Recombinant BanLec-eGFP

**Figure 1:**
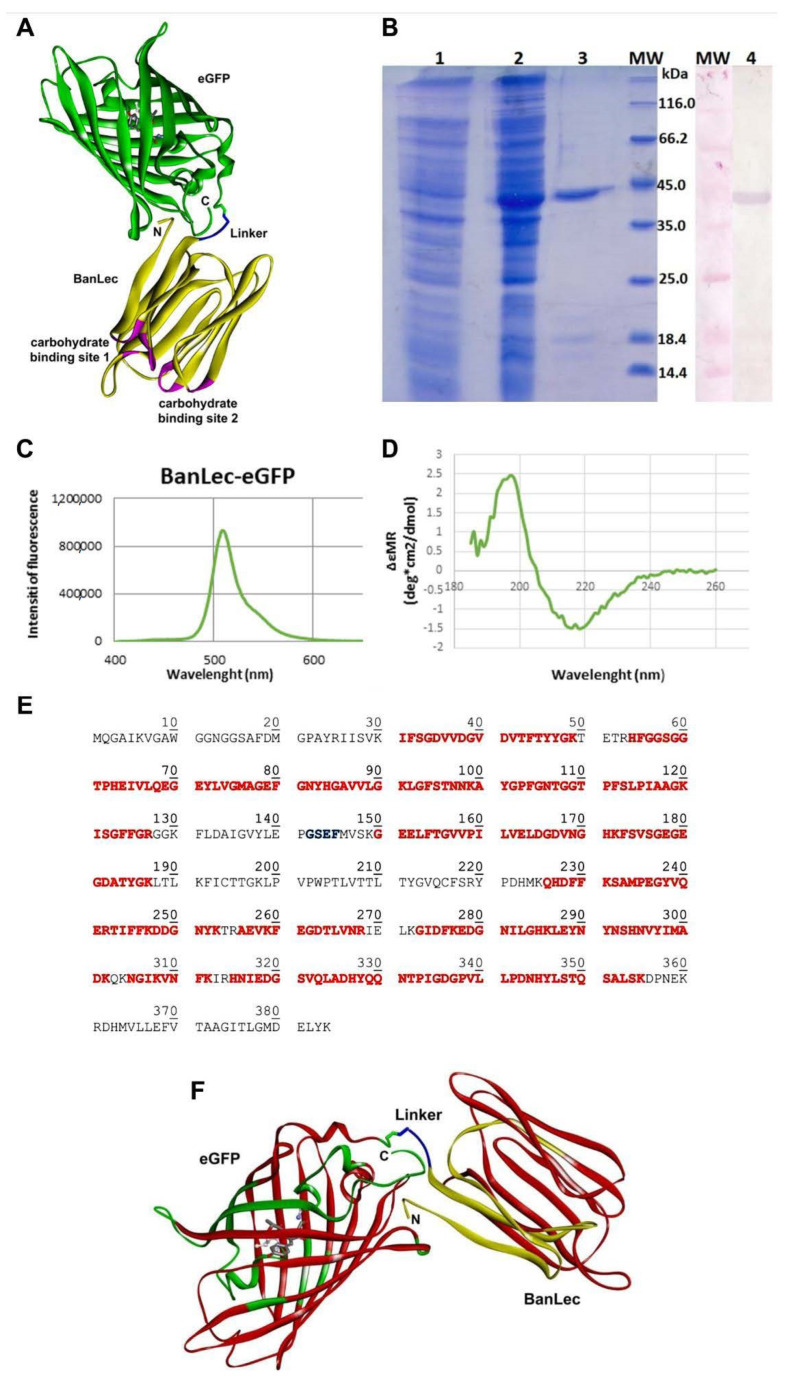
(**A**) The model structure of banana lectin-enhanced green fluorescent protein (BanLec-eGFP) chimera is rendered as a solid ribbon. BanLec is shown in yellow, eGFP in green, glycine-serine-glutamate-phenylalanine (GSEF) linker in blue and carbohydrate-binding sites in pink. The chromophore inside of eGFP is displayed as ball-and-stick. The depiction is made in Discovery Studio 19.1.0 [28]. (**B**) SDS-PAGE represents the profile of expression and purification of BanLec-eGFP (1—cell lysate before the addition of IPTG; 2—cell lysate after overnight expression; 3—BanLec-eGFP after purification; 4—detection of BanLec-eGFP by the polyclonal rabbit anti-rBanLec antibodies; MW, molecular weight marker). (**C**) Fluorescence spectra of BanLec-eGFP chimera. (**D**) Circular dichroism (CD) spectra of BanLec-eGFP in the far-ultraviolet region (185–260 nm). (**E**) The amino acid sequence of BanLec-eGFP chimera. The amino acid residues marked in red color represent the tryptic peptides that have been experimentally confirmed by the MS analysis. The linker GSEF is marked in blue. The theoretical pI = 5.66 and Mw = 41,925.32 Da for the BanLec-eGFP chimera. (**F**) The model structure of the BanLec-eGFP construct was rendered in ribbon representation. The fragments of the structure experimentally confirmed are shown in red, while the other parts of BanLec, linker, and eGFP are displayed in yellow, blue, and green, respectively. The chromophore is shown in stick rendering in a gray color. The depiction was made in Discovery Studio [28].

The model of BanLec-eGFP chimera designed in silico and fully optimized with the CHARMM22 force field [30] is depicted in Figure 1. Both carbohydrate-binding sites of BanLec are highlighted, according to [32], for further analysis and discussion. The binding site 1 consists of the GG loop (14–15), GKFLD loop (129–133) with additional S16 andV88 residues, while the binding site 2 includes the GG loop (59–60), GDVVD loop (34–38) with additional T61 and F131 residues, which interact with the bound glycans stabilizing the protein-receptor complex.

Enhanced green fluorescent protein (eGFP) is built up as the β-barrel tertiary structure with the barrel’s fluorescent chromophore. The chromophore is generated by cyclization of T66, Y67, and G68 backbone atoms, and its central position is shown in Figure 1A. On the other hand, the β-strands with loops and turns define the tertiary structure of BanLec.

BanLec-eGFP chimera structure appears to be stable over the time of simulation, mostly retaining the secondary and tertiary structure of both domains. The position of eGFP should not affect the binding capacity of BanLec for the glycans.

Gene for eGFP was cloned in the pET-23b-BL vector, and the resulting protein was expressed in *E. coli* BL21 (DE3)-pLysS. The addition of IPTG induced the expression of BanLec-eGFP. The produced recombinant protein was purified by combining affinity chromatography on a Sephadex G-75 superfine matrix and ion-exchange chromatography on an ANX column. The BanLec-eGFP chimeric protein was confirmed with Western blot using the polyclonal rabbit anti-rBanLec antibodies and alkaline phosphatase labeled anti-rabbit IgG antibodies (Figure 1B).

The yield of BanLec-eGFP after purification was about 16 mg of protein per liter of cell culture. The purified protein showed a band of approximately 42 kDa in SDS-PAGE under reducing conditions. The theoretical molecular mass of BanLec-eGFP calculated by the Compute pI/Mw tool (https://web.expasy.org/compute_pi/) was 41,925.32 Da.

### 3.2. Physical-Chemical Characterization of BanLec-eGFP

Physical-chemical characterization of BanLec-eGFP chimeric protein was performed by fluorescent spectroscopy, CD spectroscopy, and mass spectrometry. Fluorescent spectroscopy analysis revealed an emission maximum of BanLec-eGFP at 509 nm (Figure 1C).

Secondary structures of BanLec-eGFP were analyzed by CD spectroscopy in the far-UV region from 185–260 nm (Figure 1D). The protein’s secondary structures were predicted by the K2D program (http://cbdm-01.zdv.uni-mainz.de/~andrade/k2d2/), which revealed the presence of 1.64% of α-helix and 40.58% of β-strand in the BanLec-eGFP. The spectra of BanLec-eGFP showed well-defined secondary structures in the construct.

A peptide mass fingerprint was employed to confirm the primary structure of the BanLec-eGFP. MS spectrometry data were compared with theoretically obtained data using amino acid sequences (Source: https://web.expasy.org/peptide_cutter/). Peptides are shown in Table 1.

By mass analysis, more than 65% of the primary sequence was confirmed (Figure 1E). While only one amino acid V88 belonging to the binding site 1 is experimentally verified, all amino acid residues from the binding site 2 are confirmed by the MS analysis.

### 3.3. Competitive Inhibition of BanLec-eGFP Binding to Influenza Vaccine High-Mannose Glycans

**Figure 2:**
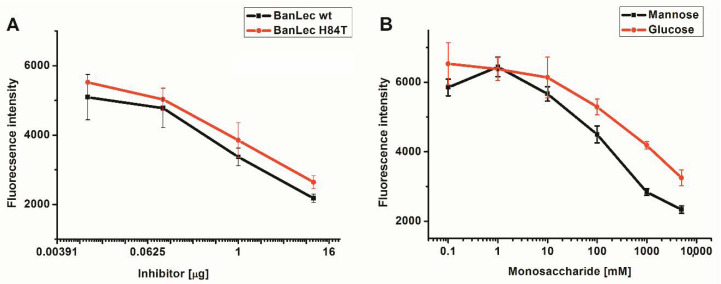
Inhibition of BanLec-eGFP binding to influenza vaccine high-mannose glycans with BanLec wt and BanLec H84T (**A**), and with monosaccharides (**B**), in fluorescence-linked lectin sorbent assay (FLLSA). The experiment was repeated three times in triplicate.

BanLec-eGFP reactivity towards high-mannose glycans present on the influenza virus vaccine was assessed by competitive FLLSA inhibition with monosaccharides mannose and glucose, and wt BanLec and BanLec H84T. This assay was done with two controls, without hemagglutinin (min) and inhibitor (max). The IC50 value for wt BanLec was 9.12 × 10^−8^ M, while IC50 for BanLec H84T was 2.35 × 10^−7^ M, indicating 2.6 higher inhibition with wt BanLec in the employed experimental settings. In accordance with our previous findings, mannose was more efficient inhibitor for BanLec carbohydrate binding (Figure 2B) than glucose [11].

### 3.4. Flow Cytometric Detection of BanLec-eGFP binding to bacteria

**Figure 3:**
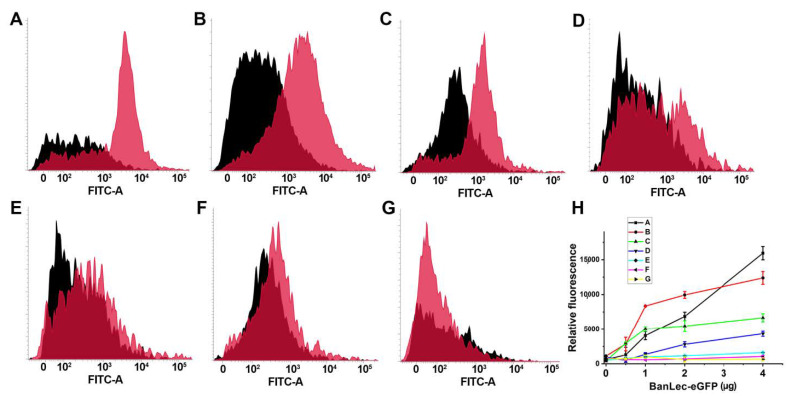
Flow cytometric analysis of the binding of BanLec-eGFP chimera to different strains of *Salmonella enterica*, subspecies *enterica.* (**A**) serovar Typhi clinical isolate 12; (**B**) serovar Typhimurium isolate 2865; (**C**) serovar Typhimurium ATCC 14028; (**D**) serovar Typhi clinical isolate 1243; (**E**) serovar Typhimurium isolate B; (**F**) serovar Enteritidis clinical isolate E; (**G**) serovar Enteritidis ATCC 13076; Black histogram—unstained bacteria; pink histogram—bacteria stained with 14 µg of BanLec-eGFP chimera; (**H**) dependence of FITC-A Median, labeled relative fluorescence on the concentration of BanLec-eGFP chimera. The strains are labeled as described above.

The binding of BanLec-eGFP was detected to certain strains of *Salmonella*. As can be seen in Figure 3, the binding was not uniform and showed differences between the strains, with especially pronounced binding to serovar Typhi isolate 12 and serovar Typhimurium isolate 2865, Figure 3A,B. The binding was also detected to *Salmonella enterica* subsp. *enterica* serovar Typhimurium ATCC 14028 and serovar Typhi isolate 1243, Figure 3C,D. Essentially no binding was detected for the other strains tested, Figure 3E–G. The dependence of relative fluorescence (FITC-A Median) on the concentration of BanLec-eGFP used to stain different strains of *Salmonella* is shown in Figure 3H.

**Figure 4:**
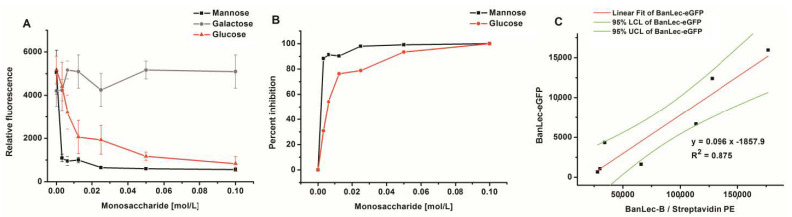
Inhibition of BanLec-eGFP binding to *Salmonella enterica*, subspecies *enterica* serovar Typhimurium isolate 2865 with different amounts of monosaccharides; panels (**A**,**B**) and correlation of staining with BanLec-eGFP with the staining with BanLec-biotin (BanLec-B) and streptavidin-phycoerythrin (PE)—panel (**C**), using flow cytometry. Each point was run in quadruplicate. Panel: (**A**) relative fluorescence; (**B**) percentage of inhibition; (**C**) correlation. For correlation experiments, 4 µg of BanLec-eGFP was used and 0.5 µg of BanLec-B with 0.2 µg streptavidin-PE (eBioscience) was used. LCL, lower confidence limit; UCL, upper confidence limit.

The binding of BanLec-eGFP was completely dependent on lectin-carbohydrate interaction as complete inhibition could be achieved with both mannose and glucose, Figure 4A,B. Galactose could not inhibit this interaction even at a high concentration of 0.1 M. Inhibition with mannose gave IC50 ≤ 1.7 mM, which was substantially lower than that obtained with glucose IC50 = 7.4 mM, Figure 4B.

In order to compare the binding of BanLec-eGFP to *Salmonella enterica* serovar Typhimurium isolate 2865 to that which would be obtained by using BanLec-B and streptavidin-PE, a correlation was made, Figure 4C. A correlation coefficient of 0.875 was obtained between the two staining procedures, with very little optimization, showing that the correlation was highly significant (*p* = 0.0019).

## 4. Discussion

In this study, recombinant BanLec-eGFP chimera was in silico designed, produced, and tested for application in the binding assay for high mannose structures present on microorganisms.

The chimera retained the secondary structures of both individual proteins and the structure was stable over the time of simulation, mostly retaining the secondary and tertiary structures of both domains, and further on as demonstrated by functional assays. The position of eGFP did not affect the binding capacity of BanLec for the glycans. This was evaluated by testing the binding of BanLec-eGFP chimera to influenza virus antigens and to different strains of *Salmonella*. These microorganisms were selected for analysis because of the presence of mannose on their surface and because of the medical relevancy.

Viruses utilize envelope proteins for receptor recognition at the entry point into the host cell. The antiviral activity of lectins is based on binding to oligosaccharides attached to the viral envelope glycoproteins and inhibiting viral fusion and entry [33].

Many viruses such as HIV, severe acute respiratory syndrome corona viruses (SARS-CoV, SARS-CoV-2), herpes simplex virus (HSV), Ebola, hepatitis B virus (HBV), hepatitis C virus (HCV), influenza, West Nile virus, dengue virus, and zika virus have glycosylated envelope proteins [33,34]. The importance of protein-sugar interactions is best illustrated by the fact that the currently used therapeutics against influenza, Tamiflu (Oseltamivir) and Relenza (Zanamivir), function as sialic acid analogs [35].

Antiviral lectins interact with glycan structures added as post-translational modifications to the viral envelope proteins [34,36]. The anti-HIV activity has been reported for the mannose-binding lectins such as the lectin from banana (BanLec), microvirin from the cyanobacterium *Microcystis aeruginosa*, scytovirin from the cyanobacteria *Scytonema varium*, lectin from snowdrop (GNA), and lectin from the red alga *Griffithsia* sp. (Griffithsin—GRFT). They have been proposed as antiviral microbicides in the prevention of HIV transmission [36,37]. Anti-influenza activity has been shown for some lectins, such as BanLec [38], cyanovirin-N [39], NICTABA (*Nicotiana tabacum* agglutinin), and UDA (*Urtica dioica* agglutinin) [40].

Various lectins have been fuzzed with fluorescent proteins as fluorescent probes for the detection of specific glycan structures present on the cell-surface or intracellular conjugates, making them versatile primary detection reagents. The neurocan–GFP was successfully employed for the detection of hyaluronan tissue distribution [24]. Fluorescently labeled *Aspergillus oryzae* lectin was a useful probe for the detection of the core fucose in N- glycans, but can be also useful vehicles for delivery of substances into the cells [26].

Vaxigrip^®^, Sanofi Pasteur IIV3, used in this study is a trivalent vaccine, containing antigens from A/H1N1, A/H3N2, and B strains, inactivated A and B split-virion influenza vaccine, (15 μg of haemagglutinin for each strain) [41]. By employing BanLec-eGFP in the competitive FLLSA, it was shown that wt BanLec exhibits higher binding for high mannose glycans present on the employed influenza vaccine than the H84T BanLec mutant, contrary to some literature data [14,38].

In order to assess the binding potential of BanLec-eGFP to highly mannosylated microorganisms, based on our experimental data and literature data, we decided to use *Sallmonella*. Previously we demonstrated that selectivity of rBanLec to fungal β-glucans can be achieved in enzyme linked lectin sorbent assay (ELLSA) [42]. The ability of BanLec to bind fungal β-glucans has been demonstrated previously by Goldstein et al. [43]. On that occasion [42] *Salmonella* was not included in the analysis. After conducting ELLSA to test the binding of BanLec to *Salmonella*, we detected approximately equal binding to that obtained with yeast cells.

*Salmonella* is a well-known bacterial pathogen that causes both food-borne gastroenteritis in humans and animals and typhoid fever. There are currently two species of *Salmonella*, with *Salmonella enterica* being medically relevant. Within the species of *S. enterica* individual subspecies and serovars have been identified. Different serovars are differentiated by the structures of their flagellum, carbohydrates, and lipopolysaccharides (LPS) [44,45]. Certain strains of *Salmonella* are known to contain mannose-rich LPS, and the binding of Mannose-binding lectin (MBL) to this LPS has been investigated [46,47,48]. Studies on the effect of MBL binding to *S. enterica* serovar Typhimurium have revealed the bactericidal properties of MBL [39,40]. The inhibitory effect of MBL on the motility of *Salmonella* occurs by affecting the energy source required for motility and the signaling pathway of chemotaxis [48].

The binding of MBL to a strain of *Salmonella* producing a mannose-rich LPS was previously demonstrated with the similar principle employed in this study, by the binding of fluorochrome conjugated anti-MBL serum [46]. The approach used in this study is a direct one, with the novel BanLec-eGFP chimera, and the results indicate that BanLec-eGFP acts in a similar fashion as MBL, which remains to be corroborated by parallel testing.

Important to note is that we have obtained differences between individual serovars and strains. In particular, serovar Typhi clinical isolate 12, showed highest binding. The tested strains from serovar Enteritidis showed no binding, while variations between strains in the serovar Typhimurium have been noticed. MBL does not bind to nonpathogenic mutants of *Salmonella enterica* serovar Montevideo that lack the mannose-rich O-polysaccharide within LPS [46] leading to the question whether mannosylation influences the pathogenicity of these organisms and in what way. The novel BanLec-eGFP chimera represents an excellent novel tool for such analysis.

In this study, we have demonstrated by inhibition experiments with monosaccharides that the binding of BanLec-eGFP to microorganisms occurs through the interaction of the lectin with the saccharide component. Moreover, we have demonstrated that there is no significant influence of the addition of GFP to the specificity of BanLec.

Each lectin molecule is characterized by affinity, specificity, and selectivity, and the comparison of different lectins can be done within basic research studies. We previously compared BanLec with other mannose binding lectins such as Concanavalin A and Lens culinaris agglutinin (LCA) and found profound differences [42]. In biotechnology, often relying on the knowledge obtained through basic research, a more targeted approach, with a specific purpose should always be applied. Based on the results presented above and other literature data, further studies including the comparison of MBL with BanLec should be performed.

In conclusion, BanLec-eGFP was produced in high yield, with retained secondary structures in both domains. The protein is stable and offers the advantage of constant emission capacity, unlike the chemically labeled lectin molecules, which increases reproducibility.

## Figures and Tables

**Table 1:** Theoretical and peptide masses of the tryptic fragments obtained by MS analysis. AA = amino acid. M = molecular weight.

[M] Determined	[M] Theoretical	Peptide Sequence	Peptide Length (-AA)
4474.01	4473.84	HNIEDGSVQLADHYQQNTPIGDGPVLLPDNHYLSTQSALSK	41
3914.708	3915.35	HFGGSGGTPHEIVLQEGEYLVGMAGEFGNYHGAVVLGK	38
2438.294	2437.73	GEELFTGVVPILVELDGDVNGHK	23
2082.038	2082.29	IFSGDVVDGVDVTFTYYGK	19
2023.074	2023.28	AYGPFGNTGGTPFSLPIAAGK	21
1973.927	1974.18	LEYNYNSHNVYIMADK	16
1542.819	1542.77	GIDFKEDGNILGHK	14
1503.769	1503.54	FSVSGEGEGDATYGK	15
1477.842	1477.75	AEVKFEGDTLVNR	13
1347.748	1347.64	TIFFKDDGNYK	11
1266.604	1266.39	SAMPEGYVQER	11
1050.576	1050.14	FEGDTLVNR	9
880.475	879.97	LGFSTNNK	8
821.416	820.90	QHDFFK	6
783.39	782.90	ISGFFGR	7
655.336	654.81	TIFFK	5
507.184	506.60	VNFK	4
430.225	430.50	NGIK	4

## Data Availability

The data that support the findings of this study are available from the corresponding author M.G.-J., upon reasonable request.
